# Diagnostic Biomarker Hsa_circ_0126218 and Functioning Prediction in Peripheral Blood Monocular Cells of Female Patients With Major Depressive Disorder

**DOI:** 10.3389/fcell.2021.651803

**Published:** 2021-05-20

**Authors:** Tianyi Bu, Zhengxue Qiao, Wenbo Wang, Xiuxian Yang, Jiawei Zhou, Lu Chen, Jiarun Yang, Jia Xu, Yanping Ji, Yini Wang, Wenxin Zhang, Yanjie Yang, Xiaohui Qiu, Yunmiao Yu

**Affiliations:** ^1^Psychology and Health Management Center, Harbin Medical University, Harbin, China; ^2^Department of Endocrinology, Peking Union Medical College Hospital, Beijing, China; ^3^Psychotherapy Department, The First Psychiatric Hospital of Harbin, Harbin, China; ^4^Department of Nursing, The Fourth Affiliated Hospital of Harbin Medical University, Harbin, China; ^5^Medical Department, The First Affiliated Hospital of Harbin Medical University, Harbin, China

**Keywords:** major depressive disorder, circular RNA, diagnostic biomarker, high throughput sequencing, bioinformatics analysis, peripheral blood mononuclear cells

## Abstract

**Introduction:**

Although major depressive diroder (MDD) has brought huge burden and challenges to society globally, effective and accurate diagnoses and treatments remain inadequate. The pathogenesis that for women are more likely to suffer from depression than men needs to be excavated as well. The function of circRNAs in pathological process of depression has not been widely investigated. This study aims to explore potential diagnostic biomarker circRNA of female patients with MDD and to investigate its role in pathogenesis.

**Methods:**

First, an expression profile of circRNAs in the peripheral blood monocular cells of MDD patients and healthy peripherals were established based on high-throughput sequencing analysis. In addition, the top 10 differentially expressed circRNAs were quantified by quantitative real-time PCR to explore diagnostic biomarkers. To further investigate the function of biomarkers in the pathogenesis of MDD, bioinformatics analysis on downstream target genes of the biomarkers was carried out.

**Results:**

There is a mass of dysregulated circRNAs in PBMCs between female MDD patients and healthy controls. Among the top 10 differentially expressed circRNAs, hsa_circ_0126218 is more feasible as a diagnostic biomarker. The expression level of hsa_circ_0126218 displayed upregulation in patients with MDD and the area under the operating characteristic curve of hsa_circ_0126218 was 0.801 (95% CI 0.7226–0.8791, *p* < 0.0001). To explain the competing endogenous RNA role of hsa_circ_0126218 in the pathogenesis of female MDD, a hsa_circ_0126218-miRNA-mRNA network was established. Gene Ontology and Kyoto Encyclopedia of Genes and Genomes pathway enrichment analyses stated that some of the enriched pathways downstream of hsa_circ_0126218 are closely related to MDD. Moreover, we established a protein-protein network to further screen out the hub genes (PIK3CA, PTEN, MAPK1, CDC42, Lyn, YES1, EPHB2, SMAD2, STAT1, and ILK). The function of hsa_circ_0126218 was refined by constructing a verified circRNA-predicted miRNA-hub gene subnetwork.

**Conclusion:**

hsa_circ_0126218 can be considered as a new female MDD biomarker, and the pathogenesis of female MDD by the downstream regulation of hsa_circ_0126218 has been predicted. These findings may help further improve the early detection, effective diagnosis, convenient monitoring of complications, precise treatment, and timely recurrence prevention of depression.

## Introduction

Major depressive disorder (MDD), as a common mental disorder, has brought huge burden and challenges to society globally (World Health Organization, 2017; Gbd 2017 Disease and Injury Incidence and Prevalence Collaborators, 2018). It not only affects the social function of patients, but also their quality of life (Smith, 2014). Even though the high prevalence and considerable risk of disability and suicide have gained great attention (Ferrari et al., 2013; Gbd 2017 Disease and Injury Incidence and Prevalence Collaborators, 2018), effective and accurate diagnoses and treatments for MDD remain inadequate (Chen et al., 2017). This may be because the diagnosis and treatment plan of MDD is still dependent on the subjective assessment of the symptomology of patients by psychiatrists and their clinical experiences. Meanwhile, there was also a close link found between duration of illness and treatment response, and shorter episodes tended to have better therapeutic efficacy (Riedel et al., 2011). Therefore, investigating high-efficient and accurate biomarkers for early diagnosis of MDD to mitigate the status is imperative.

Along with the increased advances in RNA exploration techniques and bioinformatics algorithms, increasing studies have focused on investigating the biological functions of circular RNA (circRNA). Widely recognized non-coding RNAs, circRNAs have highly tissue-specific expression patterns (Xia et al., 2016; Maass et al., 2017). circRNAs perform as a closed continuous loop by joined 3′ and 5′ ends based on covalent bonds to resist RNA exonucleases (Greene et al., 2017). The relatively stable structure, high resistance to nucleases, conservative sequence, and rich variety suggest circRNAs as ideal diagnostic biomarkers (Conn et al., 2015). In the clinic, blood-based biomarkers are preferred because of their minimally invasive collection. Biomarker circRNAs in peripheral blood monocular cells (PBMCs) have been discovered in various diseases because of their relatively high abundance and indispensable convenience, such as active tuberculosis (Huang et al., 2018), acute ischemic stroke (Dong et al., 2019), hepatocellular carcinoma (Lei et al., 2019), and Crohn’s disease (Yin et al., 2019). For patients with MDD, there are obviously many incomparable advantages to discovering diagnostic biomarker circRNAs in PBMCs. Meanwhile, circRNA could also be used as a potential therapeutic target. Circular RNA is known to play a role in miRNA sponging due to its competitive endogenous RNA mechanism. As a post-transcriptional regulator, miRNA regulates downstream gene expression based on base pairing with mRNA. That is to say, circRNA has the ability to indirectly inhibit miRNA to regulate target mRNA by competitively binding to miRNA (Hansen et al., 2013; Bezzi et al., 2017). The sponge roles they play in the pathological processes of various diseases have been widely mined, such as various cancers (Vo et al., 2019), cardiovascular disease (Aufiero et al., 2019; Liang et al., 2020), systemic lupus erythematosus (Li et al., 2018), diabetes (Haque et al., 2020), and nervous system disease (Akhter, 2018; Jia et al., 2020), to mention a few. However, few studies have been performed in the depression field, and the molecular biological functions of circRNAs in the pathogenesis of MDD are still unclear.

One main concern is that women are more vulnerable to MDD with heavier severity, higher functional impairment, younger onset age, and lower quality of life compared to men (Kornstein et al., 2000b). Furthermore, the responses of the treatment vary with gender (Kornstein et al., 2000a; Khan et al., 2005). Besides, there is a sexual dimorphism at gene expression and biomolecular mechanisms in MDD. A recent study illustrated that the overlap between MDD causing genes in women and men is quite low. In addition to gene expression and transcriptional profiles, transcription levels in MDD also assume sex-specificity (Labonté et al., 2017). Therefore, offering sex-specific diagnosis and treatment could be a new strategy for patients with MDD.

The purpose of this study was to explore potential diagnostic biomarkers of female MDD and investigate its role in pathogenesis. First, we established a circRNA expression profile in the PBMCs of three paired female MDD patients and healthy controls based on high-throughput sequencing (HTS) analysis. In addition, the top 10 differentially expressed circRNAs (DEcircRNAs) were screened for preliminary verification based on fold change. For the preliminary verification step, the top 10 DEcircRNAs in PBMCs from 20 patients with MDD and 20 healthy controls were quantified by quantitative real-time PCR (qRT-PCR) to investigate potential biomarkers. Subsequently, potential biomarkers with high diagnostic value were selected for further verification in the relatively larger cohorts (patients with MDD, *n* = 60; healthy controls, *n* = 60). Finally, bioinformatic analysis was utilized on verified biomarkers to explore the potential function in the pathogenesis of MDD.

## Materials and Methods

### Study Population and Process

In this study, 83 female patients with MDD who were admitted to the First Psychiatric Hospital of Harbin, Harbin, China between May 2018 and November 2019 were recruited. Along with 83 healthy controls, age-, gender-, and ethnic-matched healthy people who had undergone physical examination during the same period. All patients with MDD were assessed using the diagnostic criteria of the Diagnostic and Statistical Manual for Mental Illness (fifth edition, DSM-V) based on the structured interviews of two psychiatrists. And then the 32-item Hypomania Checklist (HCL-32) was utilized to rule out previous episodes of mania or hypomania. All MDD patients and healthy controls who met any of the following criteria were excluded: (1) received treatment or intervention, (2) physical or neurological diseases, (3) history of alcohol or drug abuse, (4) family history of neuropsychiatric disorders, and (5) in puberty, perimenopause, or pregnancy. The study was approved by the Ethical Committee of Harbin Medical University. Informed consent forms were signed by all the participants.

### Blood Sample Collection and RNA Extraction in PBMCs

After overnight fasting, 5 ml of peripheral blood was collected from the median cubital vein of each patient before breakfast, and then stored in EDTA (ethylenediaminetetraacetic acid) anticoagulant vacutainers. The PBMCs were isolated by density-gradient centrifugation over a human peripheral blood lymphocyte separator according to the manufacturer’s protocol. Three PBMC samples from each group were lysed with TRIzol reagent (Invitrogen, United States), and frozen at −80°C; these were later used for high-throughput sequencing analysis. For the other samples, total RNA was extracted by TRIzol reagent according to the manufacturer’s protocol. Afterward, the NanoDrop ND-2000 spectrophotometer (Thermo Fisher Scientific, Waltham, MA, United States) was used for the accurate measurement of RNA concentration. All the qualified RNA was reverse-transcribed into cDNA using random primers and Transcriptor First Strand cDNA Synthesis kits (Roche Diagnostics, Germany) according to the manufacturer’s instructions, and then frozen at −80°C for further qRT-PCR.

### High-Throughput Sequencing Analysis

To investigate the expression profile of circRNAs in MDD, three MDD and three matched healthy controls were analyzed by HTS. First, the double-stranded and single-stranded DNA present in RNA samples were removed by DNAse. The Ribo-Zero rRNA Removal Kit (Illumina, Inc.) and RNase R (Epicenter, lnc) were utilized to remove ribosomal RNA and linear RNA, respectively. Furthermore, purification was carried out using Agencourt RNAClean XP magnetic beads. All operations were performed according to the manufacturer’s protocols. Finally, sequencing was performed on the Hiseq 4000 or Hiseq X-ten platform (BGI-Shenzhen, China).

### Quantitative Real-Time PCR Analysis

In this study, the top 10 DEcircRNAs were selected for further verification. Samples were quantified by quantitative real-time PCR (qRT-PCR) using the Roche LightCycler 480II system (Roche Diagnostics, Switzerland). The reaction conditions were as follows: denaturation at 95°C for 10 min and 40 cycles of amplification including 95°C for 10 s, 55°C for 10 s, and 72°C for 10 s. RNA expression levels were normalized to human β-actin gene expression levels, and all qRT-PCR reactions were performed in triplicate. The relative expression levels of each circRNA were measured by the 2^–△^^△*Ct*^ method. The ΔCt value equaled the average of each target circRNA Ct value minus the Ct value of β-actin in the corresponding samples.

### Prediction of CircRNA-miRNA-mRNA Network

To predict the target miRNAs (microRNA) of the previously verified circRNAs, TargetScan was applied. The predicted miRNAs were further engaged in pathway enrichment analysis via the DIANA miRpath v.3 platform (Tarbase v7.0 method). The microRNAs were more likely to be related to MDD. The potential targets of these selected microRNAs were predicted by TargetScan 7.2^1^, miRDB^2^, miRWalk 3^3^, and miRPathDB v2.0^4^. Subsequently, Venny 2.1.0 was utilized to obtain the intersection of target genes in each database to enhance the reliability of the prediction.

### GO and KEGG Pathway Enrichment Analyses

To comprehensively understand the possible molecular functions of gene products, the cellular environment they locate, the biological processes they involve, and the pathogenesis of MDD, Gene Ontology (GO)^5^ function annotation and Kyoto Encyclopedia of Genes and Genomes (KEGG)^6^ pathway enrichment analysis of target genes were carried out by DAVID 6.8^7^. Screened out terms had a *P*-value of < 0.05, which showed significantly enriched functions.

### Construction of the PPI Network and Analysis of Modules

To further explain the mechanism, a protein-protein interaction (PPI) network was established by Search Tool for the Retrieval of Interacting Genes (STRING Version 11.0)^8^. Gene clusters were classified by the Molecular Complex Detection (MCODE) plug-in, and the hub genes were extracted by the Cytohubba plug-in in Cystoscope Software. Finally, the constructed circRNA-miRNA-mRNA network and PPI network were visualized using Cytoscape Software (V.3.6.1).

### Statistical Analysis

The expression differences in circRNAs among patients with MDD and healthy controls were assessed using *t*-test if the data were normally distributed. Otherwise, the Mann-Whitney U test was used. Findings with *P* < 0.05 results were considered statistically significant. Data distributions were assessed by the Shapiro-Wilk test. To evaluate the diagnostic value of the DEcircRNAs, a receiver operator characteristic (ROC) curve was established. The evaluation of diagnosis ability depended on the area under the ROC curves (AUCs). All statistical analyses were performed with GraphPad 8 software (Version 8.0.2; GraphPad Software, La Jolla, CA, United States) and R software (Version 4.0.3).

## Results

The circRNA expression levels of 160 female participants were analyzed (Supplementary Table 1). The two groups did not differ regarding mean age (MDD 42.25 ± 13.78; HC 40.41 ± 10.58). However, there were significant differences in marital status. Participants from the MDD group had a higher rate of divorce (15%) compared to the HC group (3.75%). Regarding education level and family harmony, the HC group performed better than the MDD group. Nevertheless, the proportion of the MDD group who had suffered a negative life event was significantly higher than that of the HC group.

### Identification of circRNA Expression Profile

Based on HTS, the full expression profile of circRNAs in PBMCs among three MDD patients and three healthy controls was identified. Based on threshold |log_2_FC| > 1 and *Q*-value < 0.01 criteria, 724 differentially expressed circRNAs were screened, of which 413 were upregulated and 311 were downregulated. To obtain candidate biomarkers, the top 10 DEcircRNAs were utilized for detection by qRT-PCR (Table 1). The specific primers used for qRT-PCR analysis of circRNA expression levels are listed in Table 2, except for hsa_circ_0033064, which was not designed. To identify the most likely diagnostic biomarker, a validated candidate biomarker was chosen for further validation.

**TABLE 1:** Top 10 differentially expressed circRNAs between two groups.

Gene ID	Position	log_2_FC	*P*-value	*Q*-value	Gene symbol
hsa_circ_0020959	chr11:5269501-5269717	−6.141450653	3.24E-10	6.31E-08	HBG1
hsa_circ_0005959	chr19:58805463-58806947	6.013367456	6.54E-09	0.000000984	ZNF8
hsa_circ_0033064	chr14:94582126-94583033	5.989119909	1.18E-30	8.74E-28	IFI27
hsa_circ_0006862	chr1:212216334-212220759	5.913831782	2.04E-08	0.00000277	DTL
hsa_circ_0027732	chr12:94975397-94976314	5.806916578	6.44E-08	0.00000777	TMCC3
hsa_circ_0126218	chr4:367704-367873	5.691439361	0.000000206	0.0000213	ZNF141
hsa_circ_0031578	chr14:31915242-31926680	5.691439361	0.000000206	0.0000213	C14orf126
hsa_circ_0094610	chr10:97098889-97170534	5.565908479	0.00000067	0.0000584	SORBS1
hsa_circ_0078445	chr6:158475998-158485782	5.565908479	0.00000067	0.0000584	SYNJ2
hsa_circ_0086092	chr8:145620479-145626685	5.565908479	0.00000067	0.0000584	CPSF1

**TABLE 2:** Primer used for qRT-PCR analysis of circRNAs expression levels.

Target ID	Primer sequence 5′–3′
β-actin (human)	F: GGGAAATCGTGCGTGACATT
	R: GGAACCGCTCATTGCCAAT
hsa_circ_0020959	F: TGCAGACTAGACCAGCAACA
	R: CCATGCACATCAAGGACTGG
hsa_circ_0005959	F: GGTAGCGGGAGTGATGTCTG
	R: CTTCGGAAGCTCAGGACCTAT
hsa_circ_0006862	F: TGGTCTTCACAATACCCTCTTCA
	R: CTTCATTGGCAACTGCTAGTACA
hsa_circ_0027732	F: AAGAGCCGGGTAGAACGTCAT
	R: TCAAAGTTGAGGTTGGTGTCTG
hsa_circ_0126218	F: GACGGTCCACAGATCGGAG
	R: TGACTCAGGAGCGAAAATTGTT
hsa_circ_0031578	F: TTATCCCTCAAGCTACCCTTGG
	R: AGTGCCATGTTCCACTACAAC
hsa_circ_0078445	F: ATGGACGATGGAGTGTCATCT
	R: CATGTGCCTGTCGAAAGCAG
hsa_circ_0086092	F: CGCAGCTCTACGTGTACCG
	R: GGACATGACGTTGCCAAAGAA
hsa_circ_0094610	F: ATTCCCAAGCCTTTCCATCAG
	R: TTTTGCTGTTCTCGATTGTGTTG

### Validation of the Diagnostic Biomarker

**FIGURE 1:**
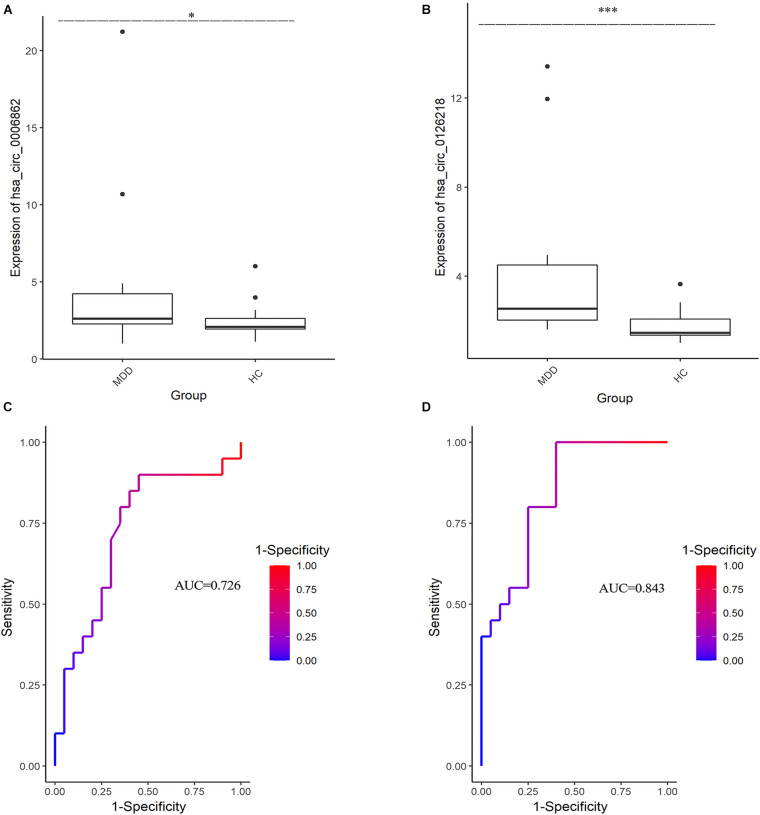
The differential expression levels and ROC curves of circRNAs in PBMCs between MDD patients (MDD, *n* = 20) and healthy controls (HC, *n* = 20) by qRT-PCR. Mann-Whitney test was utilized for data analysis. **(A)** The expression of hsa_circ_0006862 in MDD is significantly higher than that in HC. **(B)** The expression of hsa_circ_0126218 in MDD is significantly higher than that in HC. **(C)** ROC curve analysis of hsa_circ_0006862. **(D)** ROC curve analysis of hsa_circ_0126218. ROC, receiver operating characteristic.

**FIGURE 2:**
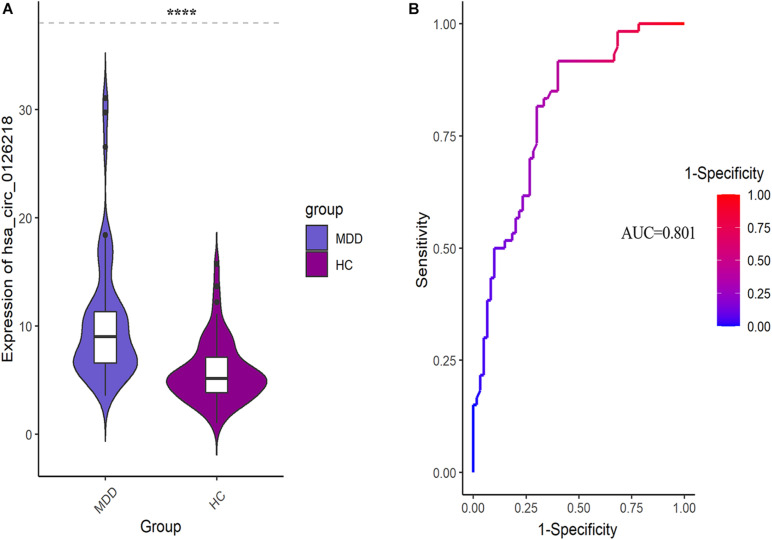
**(A)** The differential expression levels and ROC curves of hsa_circ_0126218 in PBMCs between MDD patients (MDD, *n* = 60) and healthy controls (HC, *n* = 60). **(B)** ROC curve analysis of hsa_circ_0126218. ROC, receiver operating characteristic.

The expression levels of 10 DEcirRNAs between the two groups were measured by qRT-PCR. In the preliminary validation step (MDD *n* = 20, HC *n* = 20), the levels of hsa_circ_0006862 and hsa_circ_0126218 expression were significantly higher in patients with MDD than in healthy controls (Figures 1A,B). None of the other circRNAs exhibited significant differences. The diagnostic values of these two candidate biomarkers were evaluated using ROC curves. Based on ROC curve analysis, hsa_circ_0126218 presented better diagnostic value because of the larger AUCs (AUC = 0.843, 95% CI 0.7227–0.9623, *p* = 0.0002) compared to hsa_circ_0006862 (AUC = 0.726, 95% CI 0.5633–0.8892, *p* = 0.0144) (Figures 1C,D). To further determine the actual diagnostic value of hsa_circ_0006862, a larger cohort qPT-PCR was carried out. In the further validation step of hsa_circ_0126218, there remained a significantly different expression between the two groups. The AUC of hsa_circ_0126218 was 0.801 (95% CI 0.7226–0.8791, *p* < 0.0001), suggesting that hsa_circ_0126218 has a high potential diagnostic value (Figures 2A,B).

### Interaction of CircRNA-miRNA-mRNA

**FIGURE 3:**
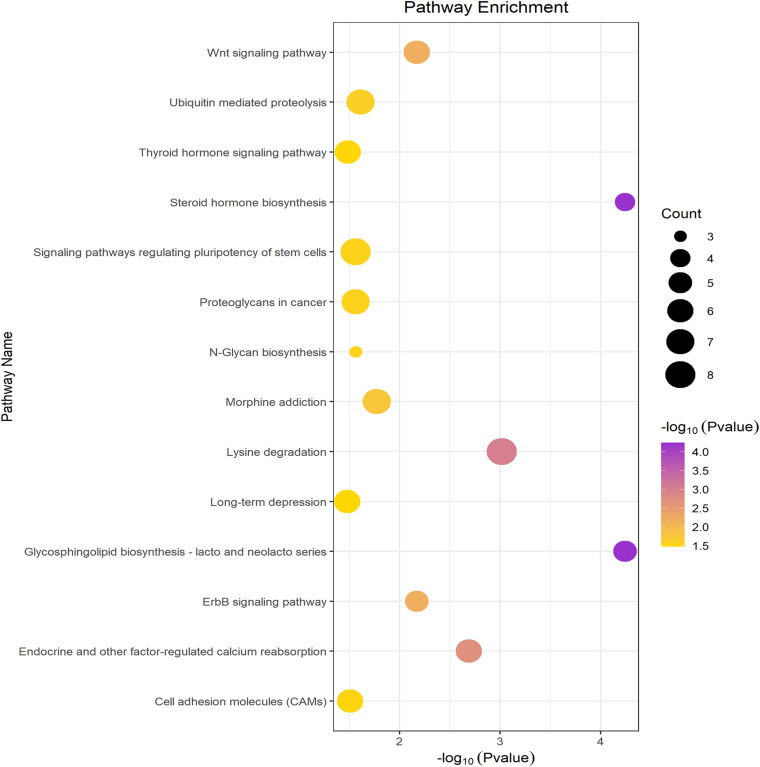
KEGG pathway analysis of top ten miRNAs.

**FIGURE 4:**
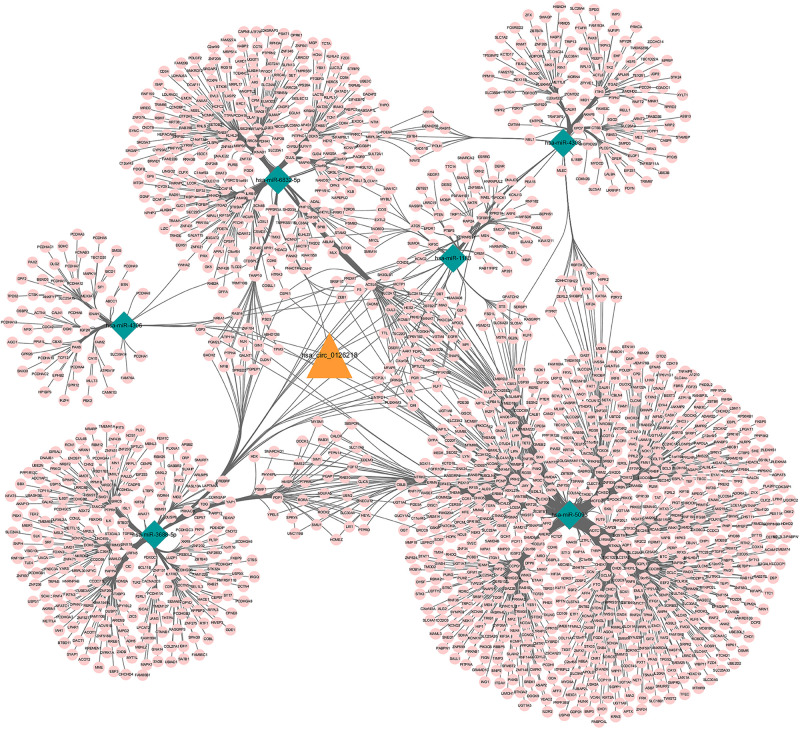
ceRNA network of circRNA-miRNA-mRNA in female major depressive disorder. The network consisting of 1 circRNA node, 6 miRNA nodes, 1541mRNA nodes. Orange color indicates circRNA, Green color presents miRNA and Pink color illustrates mRNA. circRNA, circular RNA; miRNA, microRNA.

A total of 52 miRNAs were predicted to target hsa_circ_0126218 by TargetScan. Through KEGG pathway enrichment analysis for the miRNAs that were the top 10 strongest in interacting with hsa_circ_0126218, a total of 14 pathways were demonstrated (Figure 3). Among these, the Wnt signaling pathway has already been proven to participate in the pathophysiological process of MDD (Okamoto et al., 2010; Zhou et al., 2016; Lee et al., 2019). As a result, hsa-miR-4306/hsa-miR-6832-5p/hsa-miR-1183/hsa-miR-5093/hsa-miR-4308/hsa-miR-3688-5p were concentrated in the Wnt signaling pathway. To further examine the potential downstream pathway, we selected the six miRNAs to predict target genes. A total of 1,541 target genes harbored the above six miRNAs. Subsequently, the constructed circRNA-miRNA-mRNAs network map is presented in Figure 4. hsa-miR-5093 exhibited the largest interaction network.

### GO and KEGG Pathway Enrichment Analyses

**FIGURE 5:**
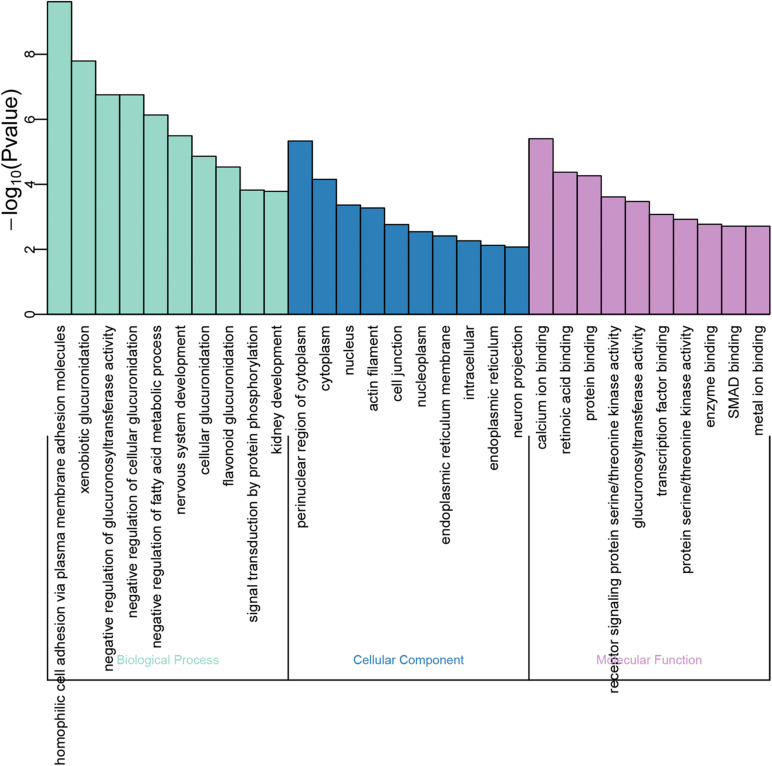
GO function annotations of the downstream target genes. The green bar chart illustrates the biological process, the blue bar chart expounds the cellular component, and the purple bar char interprets the molecular function.

**FIGURE 6:**
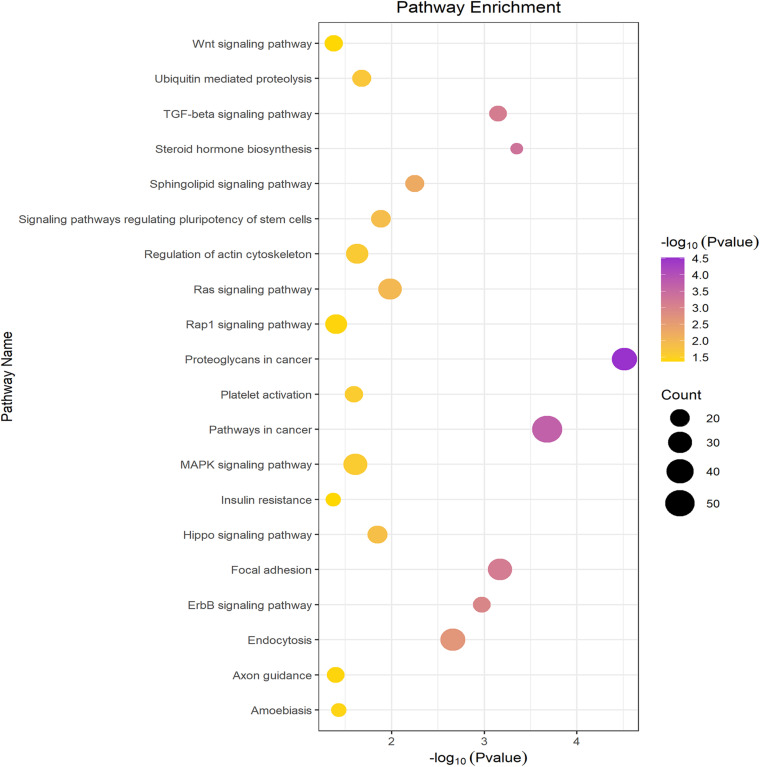
KEGG pathway analysis of the downstream target genes.

GO function annotations and KEGG pathway analysis were carried out for the target genes. Out of the GO function annotations indicated among the 61 biological process terms, the highest enriched terms were “homophilic cell adhesion via plasma membrane adhesion molecules” and “nervous system development” (*P* < 0.05). In terms of cellular components, the target mRNAs were most enriched in terms of “cytoplasm” and “nucleus” (*P* < 0.05). For molecular function, the enriched terms were “protein binding” and “metal ion binding” (*P* < 0.05) (Figure 5). According to the result of KEGG pathway analysis, 41 pathways were significantly enriched, such as “proteoglycans in cancer,” “ascorbate and aldarate metabolism,” “pathways in cancer,” “steroid hormone biosynthesis,” and “focal adhesion,” to name a few (Figure 6).

### Construction of the PPI Network and Analysis of Modules

**FIGURE 7:**
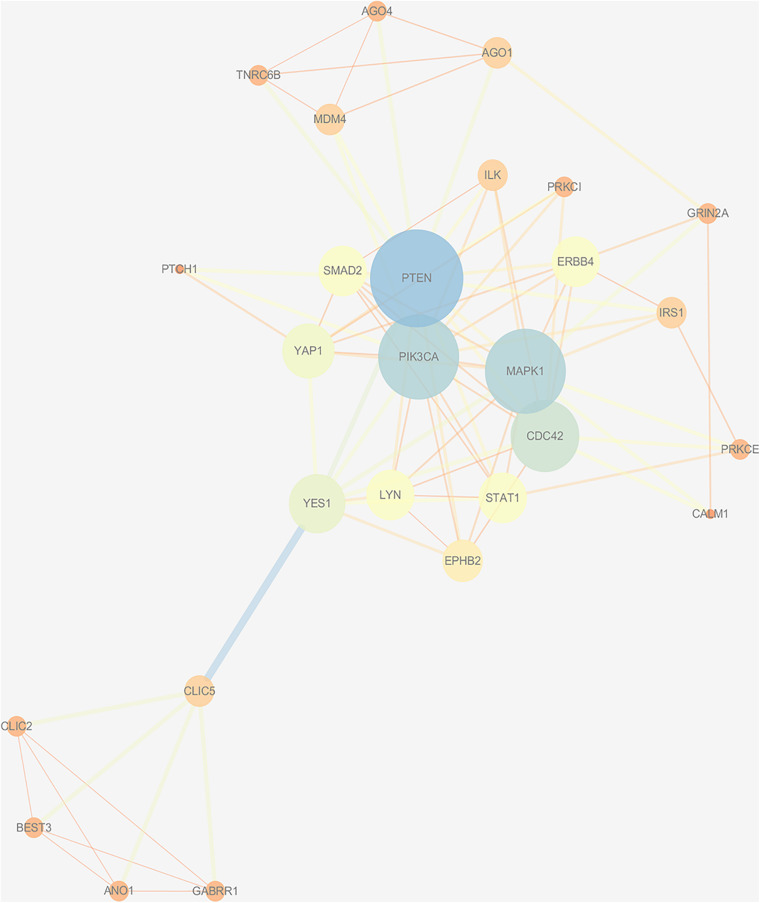
Protein-protein interaction network was visualized by Cytoscape v.3.6.1. The selected cluster identified by MCODE. The size of node depends on significance of *P*-value. The more significant *P*-value, the larger the diameter of node is. The color of the edge represents the value of combined score from 0.4 to 1, light to dark.

**FIGURE 8:**
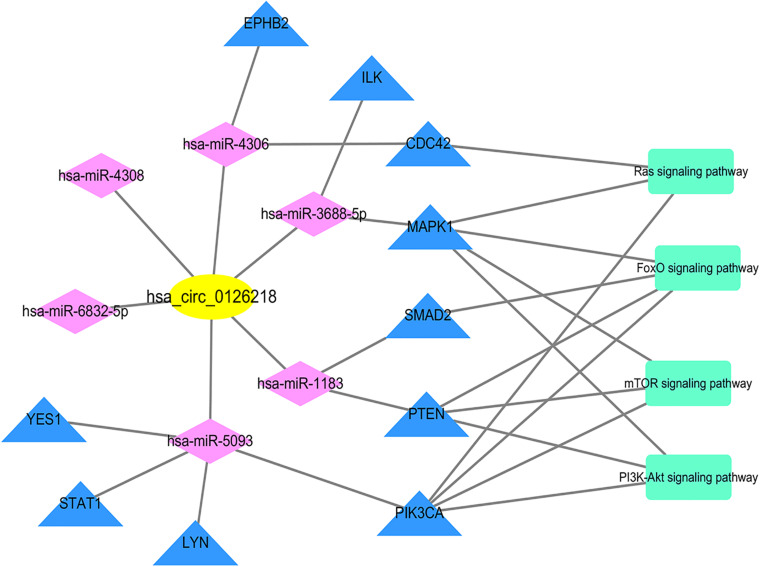
Subnetwork. The subnetwork contains 1 verified circRNA, 6 predicted miRNA and 10 screened out hub genes. Yellow color indicates circRNA, purple color presents miRNA, blue color illustrates mRNA and green color illustrates signaling pathway. circRNA, circular RNA; miRNA, microRNA.

Based on the STRING database, a PPI network with 1,541 nodes and 7,979 edges was established, in which specific information cannot be shown due to the large size of the network. The divided module was identified by MCODE from the PPI network. The significant module related with MDD contained 27 nodes and 89 edges, as shown in Figure 7. Next, the top 10 hub genes were screened out by Cytohubba based on the MCC method. They were PIK3CA, PTEN, MAPK1, CDC42, Lyn, YES1, EPHB2, SMAD2, STAT1, and ILK. Then, a verified circRNA-predicted miRNA-hub gene subnetwork was constructed (Figure 8).

## Discussion

In this study, the comprehensive understanding of MDD pathogenesis has been illustrated based on the construction of the circRNA expression profile, investigation of MDD biomarkers and prediction of therapeutic avenues and targets. Above all, a sex-specific circRNA expression profile in PBMCs from patients with MDD and healthy controls based on HTS analysis was established, and then the DEcircRNAs were screened out. The results showed that there was a difference in circRNA expression between MDD and healthy PBMCs. We performed a primary verification of the top 10 DEcircRNAs based on qRT-PCR so that a potential biomarker was obtained. A previous review indicated that the expression of circRNAs usually exhibited downregulation in cancer and some other diseases, but was upregulated in neurogenesis (Kristensen et al., 2019). In this study, 9 of the top 10 DEcircRNAs were upregulated in patients with MDD except in the case of hsa_circ_0020959. The two verified potential biomarkers were still significantly higher in patients with MDD, which is consistent with the above findings. After further verification, hsa_circ_0126218 was ultimately identified as a diagnostic biomarker for female MDD. A previous study has shown that circRNAs in peripheral blood are feasible diagnostic biomarkers due to their stable expression levels. Researchers found that there were no significant differences of each circRNA’s expression level in peripheral blood based on monitoring these circRNAs’ expression levels before and after operations of hepatocellular carcinoma (Wang et al., 2020a). The advantages of blood-based biomarkers are obvious. It may bring more opportunities for patients with MDD to regularly detect their disease-related situation. Further investigation of the ceRNA mechanism helps to understand the function of biomarker circRNA in disease pathologies. The most representative ceRNA is ciRS-7, which contains more than 70 conventional miR-7 binding sites (Hansen et al., 2013). Tang et al. proposed that elevated expression of ciRS-7 in PBMCs results in rheumatoid arthritis by reducing the inhibitory effect of miR7 on mTOR (Tang et al., 2019). Wang et al. reported that circNT5E acts as a sponge for miRNA-422a to reduce miRNA-422a regulation downstream; accordingly, this impacts proliferation, apoptosis, migration, and invasion of glioblastoma tumorigenesis cells (Wang et al., 2018). Consequently, we attempted to determine the potential mechanism of female MDD by bioinformatic analysis. To better explore the function of elevated hsa_circ_0126218 expression in the pathologies of female MDD, a circRNA-miRNA-mRNA regulatory network that provides a more intuitive way to analyze the downstream pathway was established. Five of the six miRNAs (hsa-miR-4306, hsa-miR-1183, hsa-miR-5093, hsa-miR-4308, and hsa-miR-3688-5p) involved in the ceRNA network have not been reported before.

The result of the KEGG pathway analysis pointed out that some of the pathways the genes concentrated in were closely linked to MDD, such as the “MAPK signaling pathway,” “Ras signaling pathway,” “Wnt signaling pathway,” and “insulin resistance.” A study pointed out that the MAPK signaling pathway was activated in chronic unpredictable mildly stressed mice due to the elevated p-JNK and p-p38 protein expression in the hippocampus (Su et al., 2017). Downstream of the Ras signaling pathway can extensively activate the MAPK signaling pathway, and negatively regulate nerve cell apoptosis. The role of the Ras-MAPK signaling pathway in antidepressants has drawn the attention of researchers (Wang et al., 2020b). Interestingly, a sex-specific study elucidated that different haplotypes and gene-gene interactions in the Ras-Raf-MAPK signaling pathway may more likely affect antidepressant efficacy in female patients compared to male patients (Wang et al., 2013). A recent review confirmed a popular theory that the Wnt signaling pathway takes part in depression (Tayyab et al., 2018). Another review stated the relevance between depression and insulin resistance based on meta-analysis (Kan et al., 2013). Based on the above analysis, there is more adequate evidence showing that hsa_circ_0126218 takes part in the occurrence and development of MDD.

The interactions between all the target genes were exhibited by the PPI network. The top 10 hub genes (PIK3CA, PTEN, MAPK1, CDC42, Lyn, YES1, EPHB2, SMAD2, STAT1, and ILK) were selected for further analysis. Notably, most of the above target genes are related to depression. PIK3CA, which is abundant in the nervous system, has been analyzed as a metabolite target of proteins in potential anti-depression medicine (Gao et al., 2020). A study of suicide victims in MDD expounded that the combination of dysregulated PTEN and PI3K is relevant to MDD, even though they are not concerned with suicide (Karege et al., 2011). Calabrò et al. found that MDD risk and clinical features may be attributed to the combination of alleles within MAPK1 (Seki et al., 2019). Fuchsova et al. elaborated that CDC 42 activates PAK1/PAK3 signaling to affect human depression (Fuchsova et al., 2016). There is also a close link between EphB2 inactivation and depression-like behaviors, memory impairment, and adult hippocampal neurogenesis defects (Zhen et al., 2018). Antidepressant treatment increases SMAD2 phosphorylation in the frontal cortex (Dow et al., 2005). The circRNA-predicted miRNA-hub gene sub network provides a more specific reference for further verification of pathogenesis.

Until now, there have been some unbridgeable difficulties for MDD diagnosis and treatment. Sometimes, patients’ symptoms cannot always strictly match the diagnostic criteria (Fried et al., 2016). When MDD is combined with other diseases, and the overlap symptoms enhance the difficulty of accurate diagnosis and appropriate triage (Kupfer et al., 2011; Otte et al., 2016), the risk of misdiagnosis or missed diagnosis may increase. What is worse is the inevitable reference to their private information, which increases the difficulty in acquiring the patients’ real state of illness (Schnyder et al., 2017). Moreover, the severity of MDD has no efficient objective method for appraisal. There is no doubt that precise and efficient physiological, biochemical, and pathological indicators will significantly increase the reliability and accuracy of diagnosis. Moreover, treatment efficacies need a more efficient way to monitor and evaluate patients because nearly one-third of patients still cannot get rid of MDD even though there is an ongoing development in psychotherapy and psychopharmacology (Rush et al., 2006; Thase et al., 2007). Therefore, it is valuable to find a way to detect depression early, diagnose and treat precisely, make a classification accurately, monitor complications conveniently, and prevent recurrence in a timely manner (Forsmark et al., 2016). As mentioned above, sexual dimorphism in patients with MDD is associated not only with morbidity but also at the gene expression level. The sex-specific investigation contributed to the discovery of more specific diagnostic biomarkers and mining pathological processes of MDD.

As far as we know, this study is the first of its kind that provides an expression profile of circRNA between female MDD patients and health controls by HTS, and validates the potential diagnostic biomarker function of hsa_circ_0126218 in female MDD. A blood-based test would bring more convenience and practicability in the clinic. However, some limitations need to be noted in this study. First, our study only focused on female patients, and thus could not offer a comparison between different genders. Second, the sample size was not large enough and studies on larger cohorts are needed to further verify the result. Third, the conjectural pathogenesis which was predicted by bioinformatic analysis has not been verified by experiment in this study.

In conclusion, there is a mass of differentially expressed circRNAs in PBMCs in female patients with MDD. The expression profile provided a function for further studies. hsa_circ_0126218 may be considered a new female MDD biomarker. Bioinformatic analysis suggested that hsa_circ_0126218 may play a role in the pathogenesis of female MDD by regulating its downstream target genes. Our study has attempted to highlight this diagnostic biomarker and provide a new perspective on the treatment evaluation of female MDD.

## Data Availability Statement

The datasets presented in this study can be found in online repositories. The names of the repository/repositories and accession number(s) can be found below: SRA repository, Bioproject accession number PRJNA698421.

## Ethics Statement

The studies involving human participants were reviewed and approved by the Ethical Committee of Harbin Medical University. The participants provided their written informed consent to participate in this study.

## Author Contributions

TB, YmY, and WW conducted the statistical analyses and wrote the first draft of the manuscript. XQ and JX provided expertise in MDD research. JX, YW, YJ, and JZ collected the samples. TB, WZ, and JY performed the experiment. XQ, XY, LC, and ZQ designed this study and provided expertise. YjY revised the manuscript. All authors contributed to the article and approved the submitted version.

## Conflict of Interest

The authors declare that the research was conducted in the absence of any commercial or financial relationships that could be construed as a potential conflict of interest.

## Footnotes

1 http://www.targetscan.org/vert_72/

2 http://mirdb.org/

3 http://mirwalk.umm.uni-heidelberg.de/

4 https://mpd.bioinf.uni-sb.de/overview.html

5 http://geneontology.org/

6 https://www.kegg.jp/kegg/

7 https://david.ncifcrf.gov/

8 https://string-db.org/
