# Supplementary material for: Diagnostic Biomarker Hsa_circ_0126218 and Functioning Prediction in Peripheral Blood Monocular Cells of Female Patients With Major Depressive Disorder
Source: Front Cell Dev Biol. 2021 May 20;9:651803. doi: 10.3389/fcell.2021.651803 (PMC8174117; doi:10.3389/fcell.2021.651803)
Supplement: Supplementary file 1 [file Table_1.docx]

| **Table S1.** Characteristics of the participants (n=160) | | | |
| --- | --- | --- | --- |
| Variables | HC | MDD | *P* |
| Age (years） | 40.41±10.58 | 42.45±13.78 | 0.346 |
| Site |  |  |  |
| City | 44（55.00%） | 40（50.00%） | 0.6225 |
| Downtown | 16（20.00%） | 14（17.50%） |  |
| Countryside | 20（25.00%） | 26（32.50%） |  |
| Marriage |  |  |  |
| Married | 64（80.00%） | 56（70.00%） | <0.0001* |
| Unmarried | 11（13.75%） | 12（15.00%） |  |
| Divorce | 3（3.75%） | 12（15.00%） |  |
| Widowed | 2（2.50%） | 0（0.00%） |  |
| Education |  |  |  |
| Primary school | 17（21.25%） | 18（22.50%） | 0.0022* |
| Junior high school | 10（12.50%） | 29（36.25%） |  |
| Senior high school | 28（35.00%） | 16（20.00%） |  |
| Junior college | 17（21.25%） | 11（13.75%） |  |
| Bachelor degree and above | 8（10.00%） | 6（7.50%） |  |
| Smoking |  |  |  |
| Yes | 13（16.25%） | 10（12.50%） | 0.8462 |
| No | 67（83.75%） | 70（87.50%） |  |
| Family harmony | |  |  |
| Yes | 77（96.25%） | 64（80.00%） | 0.0025* |
| No | 3（3.75%） | 16（20.00%） |  |
| Career stability |  |  |  |
| Yes | 2（2.50%） | 4（5.00%） | 0.4427 |
| No | 78（97.50%） | 76（95.00%） |  |
| Negative life events | |  |  |
| Yes | 6（7.50%） | 26（32.50%） | <0.0001* |
| No | 74（92.50%） | 54（67.50%） |  |
